# Inter­molecular hydrogen bonding in *N*-methyl-*N*′-(pyridin-2-yl)benzene-1,2-di­amine

**DOI:** 10.1107/S2056989022009173

**Published:** 2022-09-27

**Authors:** Gavin Collis, Alex Bilyk, Ueno Kazanori, Craig M. Forsyth

**Affiliations:** a CSIRO Manufacturing, Device and Engineering Systems Program, Private Bag 10, Melbourne, Victoria 3169, Australia; b School of Chemistry, Monash University, Clayton, Victoria 3800, Australia; Katholieke Universiteit Leuven, Belgium

**Keywords:** crystal structure, hydrogen bonding, dimerization, heterocycles.

## Abstract

The structure of *N*-methyl-*N*′-(pyridin-2-yl)benzene-1,2-di­amine, C_12_H_13_N_3_, at 123 K has ortho­rhom­bic (*Pna*2_1_) symmetry. The title compound displays an unexpected proton-splitting pattern when studied by ^1^H NMR spectroscopy. The X-ray crystallography analysis determined this to be caused by strong dual N—H⋯N hydrogen bonding.

## Abstract 2

Unexpected dual inter­molecular hydrogen bonding has been observed in a class of *N*-aryl-substituted *ortho*-phenyl­ene di­amine compounds. Initially detected in solution by the observation of unexplained signals in the ^1^H NMR spectrum, it was further corroborated by two-dimensional COSY NMR spectroscopy and solid-state X-ray crystallography data.

## 1. Chemical context

*ortho*-Phenyl­ene di­amine compounds are valuable precursors that have widespread use in a number of applications, especially as carbene ligands (Peris, 2018 ▸; Flanigan *et al.*, 2015 ▸; Hopkinson *et al.*, 2014 ▸; Fèvre *et al.*, 2013 ▸; Velazquez & Verpoort, 2012 ▸; Doddi *et al.*, 2019 ▸). The synthesis of *ortho*-phenyl­ene di­amine derivatives, whether it be introducing functionality on the aryl ring or the nitro­gen atom of the amine group, remains a challenge and continues to attract ongoing efforts to develop efficient synthetic routes to access a diverse library of functionalized compounds. We have been inter­ested in functionalized symmetrical and unsymmetrical *ortho*-phenyl­ene di­amine derivatives to access organometallic compounds for use in catalysis applications (Wang *et al.*, 2013 ▸) and novel aza­borole systems (Abbey & Liu, 2013 ▸; Weber, 2012 ▸, 2008 ▸; Segawa *et al.*, 2009 ▸). Although a number of symmetrical ligands, such as **I** and **II**, are readily available commercially, unsymmetrical ligands, such **III** and **IV**, are less common because the chemical routes and purification processes to access these compounds are more complicated (substituted *ortho*-phenyl­ene di­amine compounds **I**–**VI** of inter­est are shown in the scheme[Chem scheme1] below). 
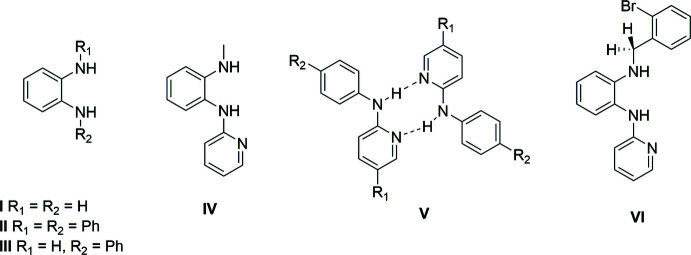


**Figure 1:**
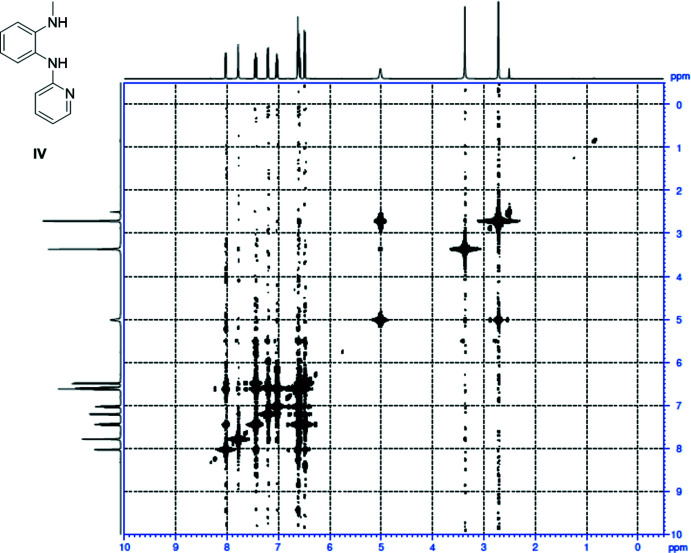
Two-dimensional COSY spectrum of *N*-methyl-*N*-phenyl-1,2-di­amino­benzene, **IV** (*d*_6_-DMSO).

Compound **IV** was synthesized by a modification of a literature procedure (Wang *et al.*, 2013 ▸). Analysis of the compound using ^1^H NMR spectroscopy to confirm the purity revealed some unexpected findings (see supporting information) when compared with similar compounds shown in the scheme[Chem scheme1]. Upon initial purification and isolation of **IV**, analysis by proton NMR in CDCl_3_ sometimes showed what seemed to be two different methyl signals, which we initially assumed was a contaminant originating from the reaction that could not be easily removed. However, analysis of the same material by ^13^C NMR spectroscopy showed a relatively simple and clean spectrum, suggesting signals for only a single compound or, if a second compound was present, the signals could be overlapping and therefore difficult to distinguish. Previously reported compounds **I** and **II** are both symmetrical and show very simple and expected signals in their respective ^1^H NMR spectra (see Figs. S1 and S2). The methyl signal of **I** was found as a singlet at 2.9 ppm and NH protons as a broad singlet at 3.0–3.5 ppm. Meanwhile, compound **II** shows the NH protons occurring as a broad singlet further downfield at 5.5–5.7 ppm, presumably caused by deshielding effects of the aryl substit­uents. Analysis of compound **IV** in CDCl_3_ shows well-defined signals that can be attributed to the aryl and pyridyl protons occurring in the downfield region between 6.3–8.3 ppm region (Fig. S3). The methyl signal occurs at 2.85 ppm, and it is inter­esting to see two different types of NH protons, a broad singlet at 4.1–4.5 ppm and a broad multiplet at 6.2–6.3 ppm. While we expect the chemical environments to be significantly different for the NH protons, we were unable to explain the multiplet-nature or coupling of these NH protons to another proton spin system. To further probe these unusual spectroscopic features, proton NMR analysis was undertaken in *d*_6_-DMSO (Fig. S4), which resulted in significant sharpening of the NH signals. The initial broad NH peak now appears as a broad multiplet around 5 ppm, and the methyl signal is split into a second order doublet, which was very unexpected. 2D COSY spectroscopy in *d*_6_-DMSO was performed on the same NMR sample (Fig. 1 ▸) and clearly showed the upfield NH multiplet at 5 ppm to be directly coupled through the nitro­gen atom with the neighbouring protons on the methyl group. On the NMR time scale, proton-to-methyl coupling through the nitro­gen atom is never reported as the NH proton is extremely labile (*i.e*. evident by very broad or even undetectable signals in proton NMR spectra) and readily undergoes facile exchange: to the best of our knowledge this type of NH proton coupling is exceptionally rare. In addition, the other downfield NH proton has sharpened further in *d*_6_-DMSO and appears as a multiplet at 6.4–6.5ppm, which suggests this NH proton may be involved in longer range coupling of the protons in the pyridyl ring. From the COSY spectrum, it appears that this NH proton is involved in long-range coupling with the pyridyl and/or phenyl protons. To understand the cause of this unexplained coupling observed in the ^1^H NMR spectra for compound **IV**, analysis by X-ray crystallographic methods was undertaken.

## 2. Structural commentary

**Figure 2:**
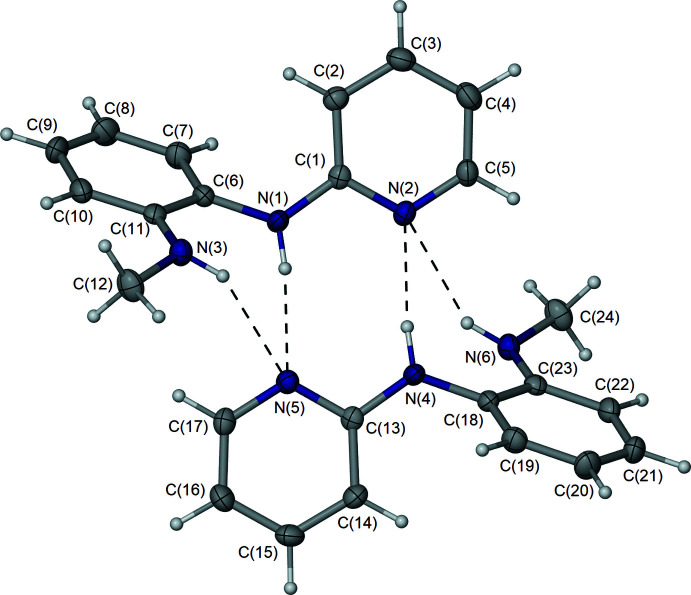
Mol­ecular diagram of the title compound, with non-hydrogen atoms represented by 50% displacement ellipsoids and hydrogen atoms as spheres of arbitrary size.

The mol­ecular structure and atom-numbering scheme of the title compound is shown in Fig. 2 ▸. The asymmetric unit comprises two independent mol­ecules assembled in a self-complementary N—H⋯N hydrogen-bonded dimer with a classical ring motif (Table 1 ▸). The overall configuration of the *N*′-(2-pyrid­yl)-benzene-1,2-di­amine core of the mol­ecule is very similar to that observed in the closely related compound, *N*-(2-bromo­benz­yl)-*N*′-(2-pyrid­yl)-benzene-1,2-di­amine (Man­jare *et al.*, 2009 ▸). The angles between the mean planes of the pyridyl and *o*-di­amino­phenyl rings are 61.80 (10) and 62.33 (10)° for mol­ecules **I** and **II**, respectively. In this configuration, the second N—H moiety on each mol­ecule is sufficiently close to the opposing pyridyl nitro­gen atom, resulting in two further but much weaker N—H⋯N inter­actions (Table 1 ▸). Notably, the equivalent *D*⋯*A* distances in *N*-(2-bromo­benz­yl)-*N*′-(2-pyrid­yl)-benzene-1,2-di­amine are approximately 0.3 Å longer. The pyridyl-amine C—N bond distances in each mol­ecule of the title compound [C1—N1 = 1.368 (3), C13—-N4 = 1.370 (3) Å] are significantly shorter than the neighbouring phenyl-amine C—N distances [C6—N1 = 1.418 (3), C18—N4 = 1.418 (3) Å], plausibly indicative of some electron delocalization associated within the pyridyl-amine fragment. Similarly, the C—N bonds associated with the second amine group display the same variation with shorter distances to the aryl-amine fragment and longer to the methyl group.

Solution and solid-state structural studies of substituted 2-aryl­amino-pyridine derivatives indicate that these mol­ecules can form two different stable conformations through rotation of the aryl ring about the C—N bond (Takasuka *et al.*, 1986 ▸). In one conformation, the pyridyl and aryl rings are not co-planar, whereas in the alternate conformation the two rings are co-planar, with an inter­molecular C—H⋯N inter­action between the pyridyl nitro­gen atoms and the 2-aminoaryl ring. The former leads to dimer formation such as observed for the parent compound 2-(phenyl­amino)­pyridine (Polamo, *et al.*, 1997 ▸) and in the current example. In contrast, the latter conformation may lead to alternate structural motifs such as 1-D catemer chains (Talja & Polamo, 2005 ▸; Polamo & Talja, 2004 ▸) or inter­actions with other functional groups (Takasuka *et al.*, 1986 ▸). The structure of *N*,*N*′-bis­(2-pyrid­yl)benzene-1,2-di­amine shows both conformations in a single mol­ecule (Gdaniec *et al.*, 2004 ▸).

Of greater inter­est is compound **VI**, which is very similar to our current compound **IV**. While compound **VI** contains the bulky 2-bromo-benzyl group attached to one amino group, it still forms the hydrogen-bonded dimer **V** complex. The reported proton NMR spectrum of **V** in CDCl_3_ (Manjare *et al.*, 2009 ▸) reveals some inter­esting features that are similar to those of compound **IV**. The methyl­ene protons on **VI** are magnetically non-equivalent whereby each proton has a different chemical shift of 4.83 and 4.41 ppm and are strongly coupled to each other. The two NH protons are also in different environments, one located downfield at 6.15 ppm as a multiplet and the other lies under a methyl­ene proton signal at 4.41 ppm, which are essentially in the same location as for compound **IV**. Inter­estingly, as compound **VI** is only analysed in CDCl_3_, the authors do not observe any coupling of the NH proton with either of the methyl­ene protons. We suspect the CH_3_–NH coupling observed in **IV** when using CDCl_3_ is less pronounced or enhanced by solvation effects than when using *d*_6_-DMSO. Nevertheless, the presence and observation of this CH_3_–NH coupling in compound **IV**, to the best of our knowledge, is rare, and in this case a result of the dual inter­molecular hydrogen bonding, occurring from the primary amino-pyridine dimer complex and secondary pyridyl and CH_3_–NH inter­action.

## 3. Supra­molecular features

**Figure 3:**
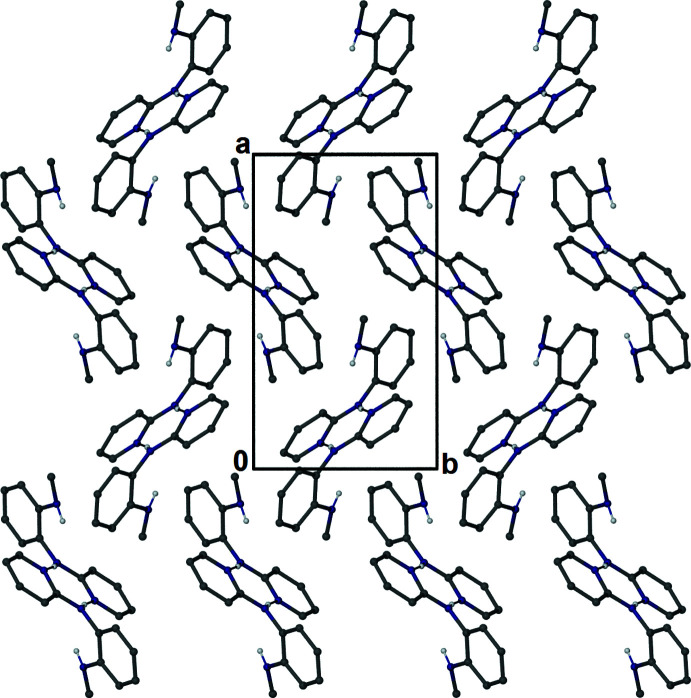
A view of the unit-cell packing, showing a single 2-D layer of hydrogen-bonded dimer mol­ecules.

The crystal packing of the title compound involves no π–π ring inter­actions [minimum *Cg*⋯*Cg* separation 4.7654 (12) Å, dihedral angle 58.68 (10)°]. There are two minor C—H⋯*Cg* inter­actions linking the dimers into a supra­molecular two-dimensional sheet lying parallel to the *ab* plane [Fig. 3 ▸; C12 ⋯*Cg*4^i^ = 3.456 (3) Å, C12—H⋯*Cg*4^i^ = 150°, H⋯*Cg*4^i^ = 2.66 Å, and C24⋯*Cg*2^ii^ = 3.565 (3) Å, C24—H⋯*Cg*2^ii^ = 152°, H⋯*Cg*2^ii^ = 2.67 Å; *Cg*2 and *Cg*4 are the centroids of rings C6–C11 and C18–C23, respectively; symmetry codes: (i) *x* − 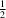 , 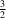 − *y*, *z*; (ii) 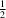 + *x*, 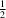 − *y*, *z*].

## 4. Database survey

A search of the Cambridge Structure Database (CSD version 5.43, November 2021; Groom *et al.*, 2016 ▸) for substituted *o*-di­amino-aryl mol­ecules with at least one 2-pyridyl substit­uent on one of the nitro­gen atoms results in only two other related compounds, *N*-(2-bromo­benz­yl)-*N*′-(2-pyrid­yl)-benzene-1,2-di­amine (Manjare *et al.*, 2009 ▸; CSD refcode RUFGIJ) and *N*,*N*′-bis­(2-pyrid­yl)benzene-1,2-di­amine (Gdaniec *et al.*, 2004 ▸; CSD refcode ARUDEW). Both of these structures show the N—H⋯N hydrogen-bonded ring motif observed in the title compound. Inter­estingly, for *N*,*N*′-bis­(2-pyrid­yl)benzene-1,2-di­amine, the second pyridyl ring is not involved in N—H⋯N hydrogen bonding. Furthermore, the related *N*,*N*′-bis­(2-pyrid­yl)benzene-1,3-di­amine and *N*,*N*′-bis­(2-pyrid­yl)benzene-1,4-di­amine compounds (Bensemann *et al.*, 2002 ▸; CSD refcodes XILPEN, XILPUD01; Wicher & Gdaniec, 2011 ▸; CSD refcode XILPUD02) show a greater complexity of N—H⋯N hydrogen-bonding motifs.

## 5. Synthesis and crystallization

*N,N′*-Dimethyl-1,2-phenyl­enedi­amine **I** and *N,N′*-diphenyl-1,2-phenyl­enedi­amine **II** were obtained from commercial sources, while compound **IV** was synthesized following modification of a literature procedure (Wang *et al.*, 2013 ▸). Inter­estingly, compounds **I** and **II** are liquids and discolour easily, possibly due to the presence of residual contaminants that may be difficult to remove completely during purification. This can make purification of the ligand difficult when the *R* groups are small (*i.e.* meth­yl). Introduction of bulky aryl groups provides materials that are crystalline and can be purified easily by chromatography and recrystallization. Compound **IV** was synthesized by methyl­ation of commercially available *N*-(pyridin-2-yl)benzene-1,2-di­amine by mod­i­fying literature conditions (Wang *et al.*, 2013 ▸) to introduce the methyl group onto the primary amine functionality. This compound was purified by chromatographic methods to afford a white solid in high purity. A suitable sample for X-ray determination was achieved by the slow diffusion of petroleum ether into a solution of **IV** dissolved in ethyl acetate.

## 6. Refinement

Crystal data, data collection and structure refinement details are summarized in Table 2 ▸. The structure was solved in the non-centrosymmetric space group *Pna*2_1_ and refined as a racemic twin [BASF 0.3 (5)]. Hydrogen atoms attached to carbon were placed in calculated positions and refined using a riding model. The hydrogen atoms of the NH groups were located in a difference-Fourier map, and freely refined.

## Supplementary Material

Crystal structure: contains datablock(s) I. DOI: 10.1107/S2056989022009173/vm2268sup1.cif

Structure factors: contains datablock(s) I. DOI: 10.1107/S2056989022009173/vm2268Isup2.hkl

Fig. S1. DOI: 10.1107/S2056989022009173/vm2268sup3.png

Fig. S2. DOI: 10.1107/S2056989022009173/vm2268sup4.png

Fig. S3. DOI: 10.1107/S2056989022009173/vm2268sup5.png

Fig. S4. DOI: 10.1107/S2056989022009173/vm2268sup6.png

CCDC reference: 2207385

Additional supporting information:  crystallographic information; 3D view; checkCIF report

## Figures and Tables

**Table 1:** Hydrogen-bond geometry (Å, °)

*D*—H⋯*A*	*D*—H	H⋯*A*	*D*⋯*A*	*D*—H⋯*A*
N4—H4⋯N2	0.90 (3)	2.11 (3)	3.001 (3)	173 (2)
N1—H1⋯N5	0.88 (3)	2.11 (3)	2.981 (3)	173 (2)
N6—H6⋯N2	0.88 (3)	2.62 (2)	3.374 (2)	145 (2)
N3—H3⋯N5	0.87 (3)	2.61 (3)	3.337 (2)	142 (2)

**Table 2:** Experimental details

Crystal data	
Chemical formula	C_12_H_13_N_3_
*M*_r_	199.25
Crystal system, space group	Orthorhombic, *P* *n* *a*2_1_
Temperature (K)	123
*a*, *b*, *c* (Å)	13.4639 (2), 7.8555 (1), 20.1288 (3)
*V* (Å^3^)	2128.94 (5)
*Z*	8
Radiation type	Cu *K*α
μ (mm^−1^)	0.60
Crystal size (mm)	0.25 × 0.13 × 0.10

Data collection	
Diffractometer	Oxford Diffraction Gemini Ultra CCD
Absorption correction	Multi-scan (*CrysAlis PRO*; Rigaku OD, 2015 ▸)
*T*_min_, *T*_max_	0.989, 1.000
No. of measured, independent and observed [*I* > 2σ(*I*)] reflections	11852, 3104, 3018
*R*_int_	0.022
(sin θ/λ)_max_ (Å^−1^)	0.596

Refinement	
*R*[*F* ^2^ > 2σ(*F* ^2^)], *wR*(*F* ^2^), *S*	0.029, 0.072, 1.06
No. of reflections	3104
No. of parameters	290
No. of restraints	1
H-atom treatment	H atoms treated by a mixture of independent and constrained refinement
Δρ_max_, Δρ_min_ (e Å^−3^)	0.12, −0.16
Absolute structure	Refined as an inversion twin
Absolute structure parameter	0.3 (5)

Computer programs: *CrysAlis PRO* (Rigaku OD, 2015 ▸), *SHELXT2018/3* (Sheldrick, 2015*a* ▸), *SHELXL2018/3* (Sheldrick, 2015*b* ▸), *X-SEED* (Barbour, 2001 ▸), *publCIF* (Westrip, 2010 ▸).

## supplementary crystallographic information

### Crystal data

**Table:**

C_12_H_13_N_3_	*D*_x_ = 1.243 Mg m^−^^3^
*M**_r_* = 199.25	Cu *K*α radiation, λ = 1.54178 Å
Orthorhombic, *P**n**a*2_1_	Cell parameters from 6421 reflections
*a* = 13.4639 (2) Å	θ = 5.5–66.8°
*b* = 7.8555 (1) Å	µ = 0.60 mm^−^^1^
*c* = 20.1288 (3) Å	*T* = 123 K
*V* = 2128.94 (5) Å^3^	Prism, colourless
*Z* = 8	0.25 × 0.13 × 0.10 mm
*F*(000) = 848	

### Data collection

**Table:**

Oxford Diffraction Gemini Ultra CCD diffractometer	3104 independent reflections
Radiation source: fine focus sealed tube	3018 reflections with *I* > 2σ(*I*)
Detector resolution: 10.3389 pixels mm^-1^	*R*_int_ = 0.022
ω scans	θ_max_ = 66.8°, θ_min_ = 6.1°
Absorption correction: multi-scan (CrysAlisPro; Rigaku OD, 2015)	*h* = −16→15
*T*_min_ = 0.989, *T*_max_ = 1.000	*k* = −9→8
11852 measured reflections	*l* = −19→23

### Refinement

**Table:**

Refinement on *F*^2^	Hydrogen site location: mixed
Least-squares matrix: full	H atoms treated by a mixture of independent and constrained refinement
*R*[*F*^2^ > 2σ(*F*^2^)] = 0.029	*w* = 1/[σ^2^(*F*_o_^2^) + (0.0408*P*)^2^ + 0.2845*P*] where *P* = (*F*_o_^2^ + 2*F*_c_^2^)/3
*wR*(*F*^2^) = 0.072	(Δ/σ)_max_ < 0.001
*S* = 1.06	Δρ_max_ = 0.12 e Å^−^^3^
3104 reflections	Δρ_min_ = −0.16 e Å^−^^3^
290 parameters	Absolute structure: Refined as an inversion twin
1 restraint	Absolute structure parameter: 0.3 (5)

### Special details

**Table:**

Geometry. All esds (except the esd in the dihedral angle between two l.s. planes) are estimated using the full covariance matrix. The cell esds are taken into account individually in the estimation of esds in distances, angles and torsion angles; correlations between esds in cell parameters are only used when they are defined by crystal symmetry. An approximate (isotropic) treatment of cell esds is used for estimating esds involving l.s. planes.
Refinement. Refined as a 2-component inversion twin.

### Fractional atomic coordinates and isotropic or equivalent isotropic displacement parameters (Å^2^)

**Table:**

	*x*	*y*	*z*	*U*_iso_*/*U*_eq_	
N1	0.05732 (12)	0.4385 (2)	0.17463 (9)	0.0216 (4)	
N2	0.17895 (11)	0.6413 (2)	0.18021 (8)	0.0197 (3)	
N3	−0.12863 (12)	0.4264 (2)	0.23539 (10)	0.0218 (4)	
N4	0.20213 (12)	0.5712 (2)	0.32597 (9)	0.0215 (4)	
N5	0.08245 (12)	0.3655 (2)	0.31899 (9)	0.0191 (3)	
N6	0.38962 (12)	0.5723 (2)	0.26687 (9)	0.0213 (4)	
C1	0.10475 (13)	0.5720 (2)	0.14465 (10)	0.0182 (4)	
C2	0.07758 (14)	0.6353 (3)	0.08167 (10)	0.0220 (4)	
H2	0.024921	0.584736	0.057237	0.026*	
C3	0.12892 (16)	0.7717 (3)	0.05649 (14)	0.0276 (5)	
H3A	0.111761	0.816520	0.014189	0.033*	
C4	0.20612 (16)	0.8445 (3)	0.09273 (12)	0.0286 (5)	
H4A	0.242660	0.938570	0.075915	0.034*	
C5	0.22743 (15)	0.7749 (3)	0.15392 (12)	0.0229 (5)	
H5	0.279645	0.824378	0.179112	0.027*	
C6	−0.01999 (14)	0.3405 (2)	0.14590 (10)	0.0200 (4)	
C7	−0.00450 (17)	0.2503 (3)	0.08767 (13)	0.0273 (5)	
H7	0.058430	0.255938	0.066431	0.033*	
C8	−0.07908 (18)	0.1521 (3)	0.05989 (12)	0.0325 (5)	
H8	−0.068144	0.092462	0.019500	0.039*	
C9	−0.17014 (17)	0.1423 (3)	0.09204 (12)	0.0316 (5)	
H9	−0.221668	0.074427	0.073659	0.038*	
C10	−0.18668 (15)	0.2303 (3)	0.15057 (13)	0.0244 (5)	
H10	−0.249258	0.221028	0.172068	0.029*	
C11	−0.11291 (14)	0.3325 (2)	0.17851 (10)	0.0192 (4)	
C12	−0.21406 (16)	0.3957 (3)	0.27773 (12)	0.0297 (5)	
H12A	−0.274707	0.430287	0.254431	0.045*	
H12B	−0.207321	0.461959	0.318748	0.045*	
H12C	−0.217817	0.274311	0.288601	0.045*	
C13	0.15406 (13)	0.4378 (2)	0.35580 (10)	0.0179 (4)	
C14	0.17762 (14)	0.3787 (3)	0.41966 (11)	0.0235 (4)	
H14	0.228036	0.432290	0.445205	0.028*	
C15	0.12622 (17)	0.2417 (3)	0.44443 (14)	0.0281 (5)	
H15	0.141481	0.198837	0.487358	0.034*	
C16	0.05185 (16)	0.1657 (3)	0.40675 (12)	0.0277 (5)	
H16	0.015320	0.071136	0.423112	0.033*	
C17	0.03327 (15)	0.2328 (3)	0.34494 (12)	0.0230 (5)	
H17	−0.017626	0.181957	0.318960	0.028*	
C18	0.28032 (14)	0.6664 (2)	0.35511 (10)	0.0196 (4)	
C19	0.26463 (16)	0.7599 (3)	0.41236 (13)	0.0252 (5)	
H19	0.200977	0.758902	0.432723	0.030*	
C20	0.34067 (17)	0.8553 (3)	0.44053 (11)	0.0307 (5)	
H20	0.329472	0.918276	0.480110	0.037*	
C21	0.43291 (16)	0.8573 (3)	0.41021 (12)	0.0288 (5)	
H21	0.485315	0.922046	0.429208	0.035*	
C22	0.44960 (16)	0.7658 (3)	0.35234 (13)	0.0240 (5)	
H22	0.513234	0.769323	0.331998	0.029*	
C23	0.37397 (14)	0.6684 (2)	0.32350 (10)	0.0191 (4)	
C24	0.47575 (16)	0.6013 (3)	0.22502 (12)	0.0289 (5)	
H24A	0.470921	0.530363	0.185112	0.043*	
H24B	0.536175	0.571693	0.249577	0.043*	
H24C	0.478297	0.721543	0.212130	0.043*	
H1	0.0701 (18)	0.421 (3)	0.2170 (14)	0.027 (7)*	
H3	−0.0774 (19)	0.465 (3)	0.2568 (13)	0.026 (6)*	
H4	0.1913 (19)	0.585 (3)	0.2824 (16)	0.034 (7)*	
H6	0.3354 (18)	0.540 (3)	0.2465 (12)	0.022 (6)*	

### Atomic displacement parameters (Å^2^)

**Table:**

	*U*^11^	*U*^22^	*U*^33^	*U*^12^	*U*^13^	*U*^23^
N1	0.0207 (8)	0.0279 (9)	0.0160 (9)	−0.0072 (7)	−0.0018 (7)	0.0013 (7)
N2	0.0170 (7)	0.0225 (7)	0.0194 (8)	−0.0011 (6)	0.0000 (6)	−0.0029 (7)
N3	0.0195 (8)	0.0224 (9)	0.0236 (9)	−0.0020 (6)	0.0016 (7)	−0.0022 (7)
N4	0.0197 (8)	0.0283 (9)	0.0165 (9)	−0.0069 (7)	−0.0027 (7)	0.0031 (8)
N5	0.0164 (7)	0.0216 (8)	0.0192 (8)	−0.0005 (6)	0.0009 (6)	−0.0004 (7)
N6	0.0181 (8)	0.0222 (8)	0.0235 (9)	−0.0027 (6)	0.0016 (7)	−0.0012 (8)
C1	0.0161 (8)	0.0197 (9)	0.0187 (10)	0.0009 (7)	0.0028 (7)	−0.0035 (8)
C2	0.0215 (9)	0.0246 (10)	0.0197 (10)	0.0011 (8)	−0.0004 (8)	−0.0016 (9)
C3	0.0329 (12)	0.0271 (11)	0.0226 (12)	0.0000 (9)	−0.0001 (9)	0.0048 (10)
C4	0.0341 (11)	0.0241 (10)	0.0278 (12)	−0.0064 (9)	0.0034 (9)	0.0025 (9)
C5	0.0223 (9)	0.0237 (9)	0.0227 (13)	−0.0056 (8)	0.0021 (8)	−0.0027 (9)
C6	0.0204 (9)	0.0208 (9)	0.0187 (10)	−0.0035 (7)	−0.0011 (8)	0.0030 (8)
C7	0.0295 (11)	0.0301 (12)	0.0222 (13)	−0.0054 (9)	0.0028 (9)	−0.0011 (9)
C8	0.0454 (13)	0.0309 (12)	0.0212 (11)	−0.0094 (10)	−0.0003 (10)	−0.0052 (10)
C9	0.0385 (12)	0.0300 (11)	0.0263 (12)	−0.0141 (9)	−0.0099 (10)	0.0005 (10)
C10	0.0216 (9)	0.0251 (10)	0.0265 (13)	−0.0049 (8)	−0.0026 (9)	0.0056 (9)
C11	0.0207 (9)	0.0169 (9)	0.0200 (10)	0.0004 (7)	−0.0033 (8)	0.0038 (8)
C12	0.0263 (11)	0.0295 (11)	0.0333 (13)	−0.0034 (9)	0.0102 (9)	−0.0011 (10)
C13	0.0149 (8)	0.0203 (9)	0.0185 (10)	0.0010 (7)	0.0029 (7)	−0.0024 (8)
C14	0.0215 (9)	0.0266 (10)	0.0224 (10)	−0.0012 (8)	−0.0046 (8)	0.0004 (9)
C15	0.0349 (13)	0.0280 (11)	0.0213 (12)	−0.0011 (9)	−0.0042 (9)	0.0061 (9)
C16	0.0306 (10)	0.0227 (10)	0.0299 (12)	−0.0056 (8)	0.0000 (9)	0.0044 (9)
C17	0.0217 (10)	0.0204 (9)	0.0270 (14)	−0.0024 (8)	−0.0013 (8)	−0.0033 (9)
C18	0.0187 (9)	0.0220 (9)	0.0181 (10)	−0.0036 (7)	−0.0030 (8)	0.0031 (8)
C19	0.0255 (10)	0.0289 (12)	0.0213 (13)	−0.0036 (8)	0.0035 (9)	0.0002 (9)
C20	0.0373 (12)	0.0349 (11)	0.0198 (11)	−0.0098 (10)	0.0007 (9)	−0.0060 (10)
C21	0.0296 (11)	0.0317 (11)	0.0252 (11)	−0.0123 (8)	−0.0050 (9)	0.0007 (10)
C22	0.0193 (9)	0.0264 (10)	0.0264 (13)	−0.0048 (8)	−0.0028 (8)	0.0045 (9)
C23	0.0200 (9)	0.0176 (9)	0.0197 (10)	0.0001 (7)	−0.0014 (8)	0.0044 (8)
C24	0.0277 (10)	0.0276 (11)	0.0315 (12)	−0.0015 (9)	0.0111 (9)	−0.0005 (10)

### Geometric parameters (Å, º)

**Table:**

N1—C1	1.368 (3)	C6—C7	1.386 (3)
N1—C6	1.418 (3)	C6—C11	1.414 (3)
N2—C5	1.344 (3)	C7—C8	1.384 (3)
N2—C1	1.344 (2)	C8—C9	1.389 (3)
N3—C11	1.378 (3)	C9—C10	1.384 (4)
N3—C12	1.452 (3)	C10—C11	1.395 (3)
N4—C13	1.370 (3)	C13—C14	1.403 (3)
N4—C18	1.418 (3)	C14—C15	1.373 (3)
N5—C17	1.341 (3)	C15—C16	1.391 (3)
N5—C13	1.342 (3)	C16—C17	1.374 (3)
N6—C23	1.384 (3)	C18—C19	1.383 (3)
N6—C24	1.451 (3)	C18—C23	1.412 (3)
C1—C2	1.410 (3)	C19—C20	1.390 (3)
C2—C3	1.372 (3)	C20—C21	1.384 (3)
C3—C4	1.393 (3)	C21—C22	1.387 (4)
C4—C5	1.378 (3)	C22—C23	1.400 (3)
			
C1—N1—C6	125.37 (18)	N3—C11—C10	122.23 (18)
C5—N2—C1	117.88 (18)	N3—C11—C6	119.83 (17)
C11—N3—C12	121.38 (17)	C10—C11—C6	117.92 (19)
C13—N4—C18	124.93 (17)	N5—C13—N4	114.92 (17)
C17—N5—C13	117.96 (18)	N5—C13—C14	121.87 (18)
C23—N6—C24	120.93 (17)	N4—C13—C14	123.21 (18)
N2—C1—N1	115.00 (17)	C15—C14—C13	118.6 (2)
N2—C1—C2	121.89 (18)	C14—C15—C16	120.1 (2)
N1—C1—C2	123.09 (18)	C17—C16—C15	117.4 (2)
C3—C2—C1	118.5 (2)	N5—C17—C16	124.1 (2)
C2—C3—C4	120.2 (2)	C19—C18—C23	120.39 (18)
C5—C4—C3	117.4 (2)	C19—C18—N4	120.74 (18)
N2—C5—C4	124.1 (2)	C23—C18—N4	118.84 (17)
C7—C6—C11	120.20 (18)	C18—C19—C20	120.9 (2)
C7—C6—N1	120.80 (18)	C21—C20—C19	119.2 (2)
C11—C6—N1	118.97 (17)	C20—C21—C22	120.64 (19)
C8—C7—C6	121.2 (2)	C21—C22—C23	120.9 (2)
C7—C8—C9	118.9 (2)	N6—C23—C22	121.96 (19)
C10—C9—C8	120.7 (2)	N6—C23—C18	120.07 (17)
C9—C10—C11	121.1 (2)	C22—C23—C18	117.95 (19)

### Hydrogen-bond geometry (Å, º)

**Table:**

*D*—H···*A*	*D*—H	H···*A*	*D*···*A*	*D*—H···*A*
N4—H4···N2	0.90 (3)	2.11 (3)	3.001 (3)	173 (2)
N1—H1···N5	0.88 (3)	2.11 (3)	2.981 (3)	173 (2)
N6—H6···N2	0.88 (3)	2.62 (2)	3.374 (2)	145 (2)
N3—H3···N5	0.87 (3)	2.61 (3)	3.337 (2)	142 (2)
