# Supplementary figures and images for: Inter­molecular hydrogen bonding in N-methyl-N′-(pyridin-2-yl)benzene-1,2-di­amine
Source: Acta Crystallogr E Crystallogr Commun. 2022 Sep 27;78(Pt 10):1048–51. doi: 10.1107/S2056989022009173 (PMC9535833; doi:10.1107/S2056989022009173)

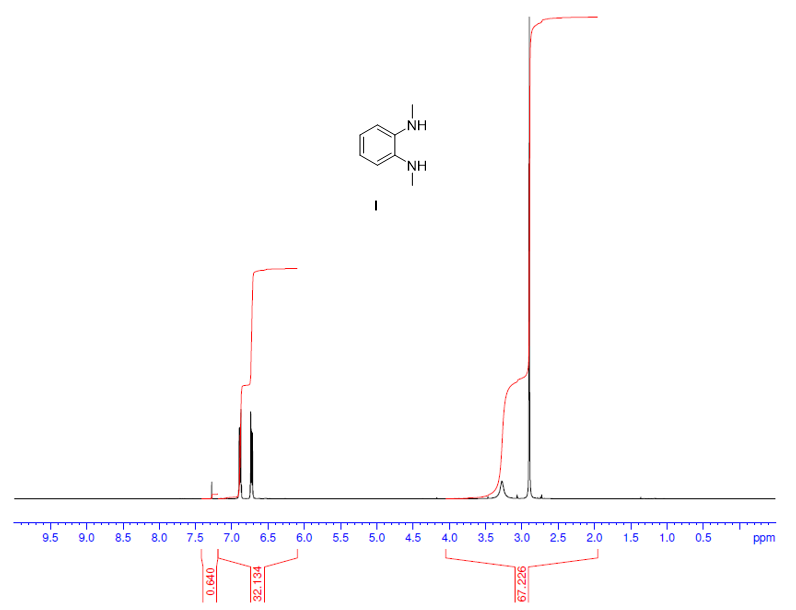

Supplement: Supplementary file 3 [file e-78-01048-sup3.png]

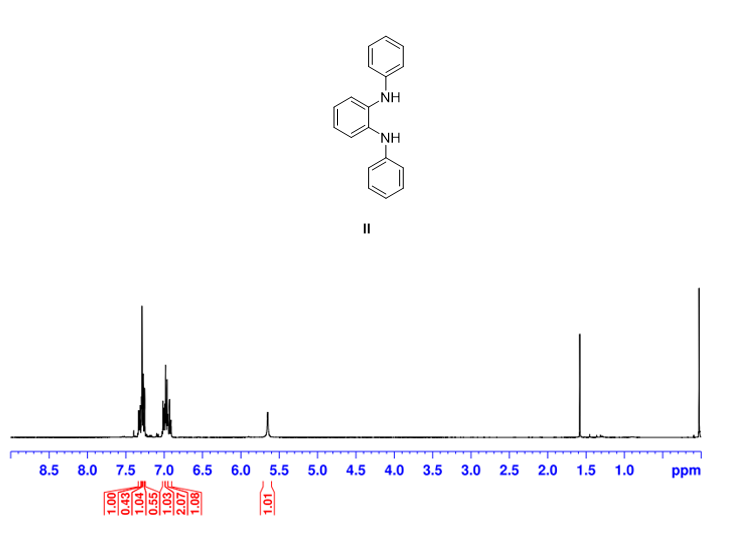

Supplement: Supplementary file 4 [file e-78-01048-sup4.png]

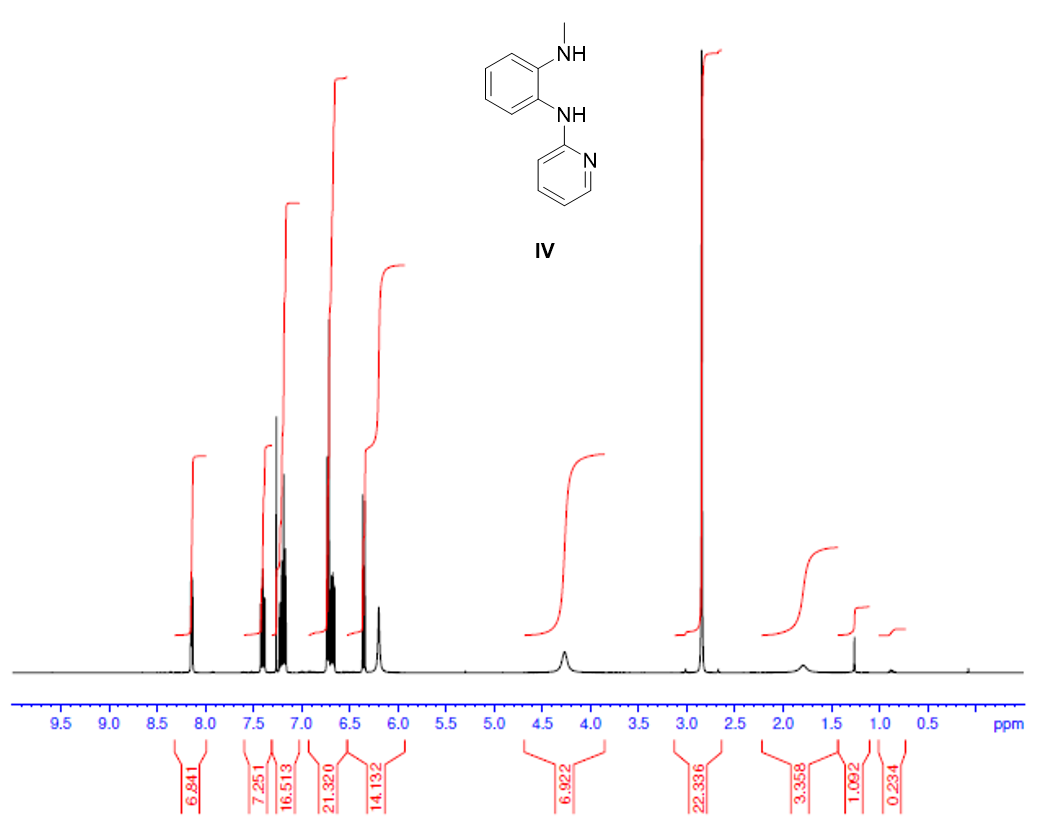

Supplement: Supplementary file 5 [file e-78-01048-sup5.png]

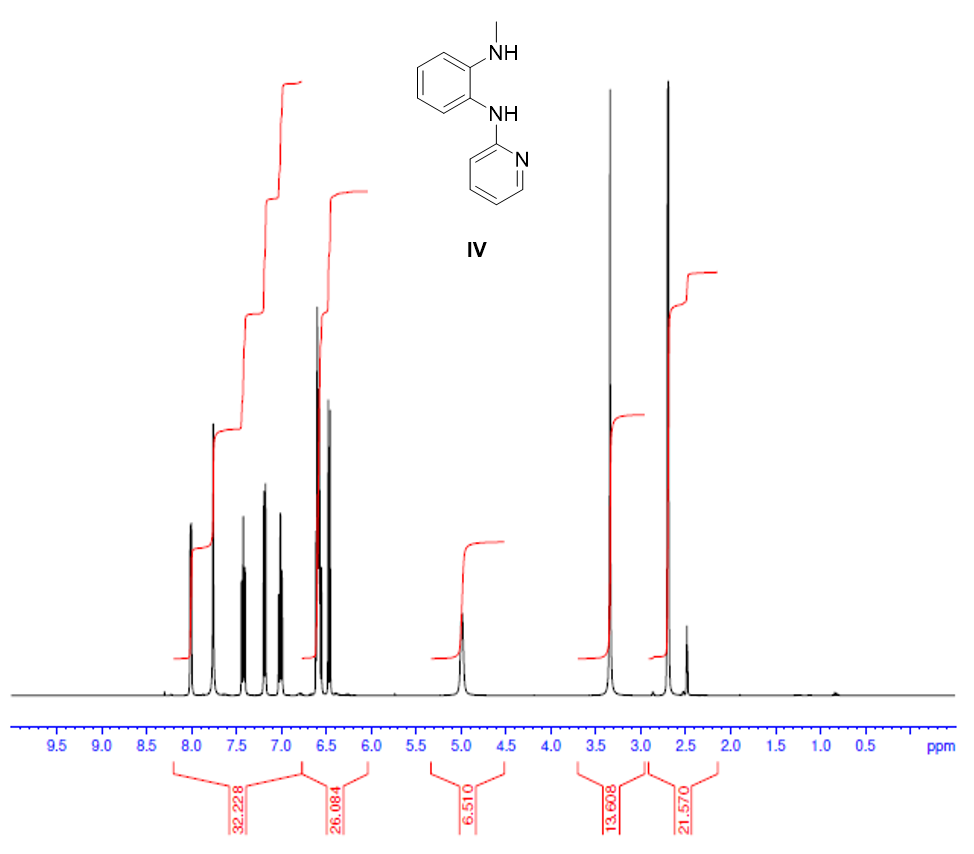

Supplement: Supplementary file 6 [file e-78-01048-sup6.png]
